# Is Computed Tomography Necessary for Diagnostic Workup in Displaced Pediatric Medial Epicondyle Fractures?

**DOI:** 10.3390/diagnostics10110957

**Published:** 2020-11-17

**Authors:** Sungmin Kim, Hyun Woo Kim, Kun-Bo Park, Kee-Bum Hong, Hoon Park

**Affiliations:** 1 Department of Orthopaedic Surgery, Chonnam National University Hospital and Medical School, College of Medicine, Gwangju 61469, Korea; kimsum83@gmail.com; 2 Division of Pediatric Orthopaedic Surgery, Severance Children’s Hospital, Yonsei University College of Medicine, Seoul 03722, Korea; pedhkim@yuhs.ac (H.W.K.); pedoskbp@yuhs.ac (K.-B.P.); 3 Department of Orthopaedic Surgery, Gangnam Severance Hospital, Yonsei University College of Medicine, Seoul 06273, Korea; icefroast@naver.com

**Keywords:** medial epicondyle, fracture, computed tomography

## Abstract

This study aimed to compare the treatment outcomes and complications between operatively and nonoperatively treated medial epicondyle fractures with displacement of >5 mm as accurately measured on three-dimensional computed tomography (3D CT). We retrospectively reviewed 77 patients who had isolated medial epicondylar fractures with displacement of >5 mm. The mean age at injury was 11.4 years. Patients were assigned to one of two groups: 21 patients treated nonoperatively and 56 patients treated surgically. Additionally, patients treated operatively were divided into two subgroups according to fixation method; 31 patients underwent internal fixation with K-wires and 25 patients underwent internal fixation with a screw. Radiological and functional outcomes were compared among the three groups. Although the bony union rate was significantly higher in patients treated operatively compared to patients treated non-operatively (96.4% vs. 23.8%, *p* < 0.001), there were no significant differences in functional outcomes between the two groups. In the nonoperative group, three patients underwent osteosynthesis for symptomatic nonunion. There were no significant differences in radiological and functional outcomes between the two subgroups divided by fixation method. In a pediatric medial epicondylar fracture with a displacement of >5 mm as accurately measured on 3D CT, despite the difference in union rate, there was no difference in functional outcomes between operative and nonoperative treatment. Performing CT only to measure the fracture displacement in obviously displaced medial epicondylar fracture is not considered as a part of the “necessary” diagnostic workups.

## 1. Introduction

Although no consensus exists in the literature as to the amount of fracture displacement that warrants surgical intervention, 5 mm of displacement has often been recommended as the threshold for surgical intervention [1,2,3,4,5]. However, literature is scarce regarding the outcomes of operative and nonoperative treatments for medial epicondylar fractures with a significant displacement of >5 mm. Most previous comparative studies included patients with even minimal displaced fractures of <5 mm [6,7,8,9]. To the best of our knowledge, there is only one previous comparative study in the literature addressing operative versus nonoperative management of medial epicondyle fractures with greater than 5 mm of displacement [5].

The clinical decision to operate continues to rely heavily on the amount of displacement among pediatric orthopedic surgeons [10,11]. Until now, all previous comparative studies investigating the treatment results measured fracture displacement by using plain radiograph [2,5,6,7,8,9]. However, standard radiographs are not sufficient to measure anterior or medial displacement of fracture fragment [12], and Pappas et al. demonstrated the inconsistency and inaccuracy of the measurement of displacement on radiographs [13]. It was found that three-dimensional computed tomography (3D CT) was the most accurate method to assess true displacement [12], and was relevant for the treatment decision [14]. Nevertheless, there has been no clinical study investigating treatment outcome based on the amount of fracture displacement using 3D CT. 

Therefore, this study aimed to compare the treatment outcomes and complications between operatively and nonoperatively treated isolated medial epicondyle fractures with displacement of >5 mm, as accurately measured by 3D CT, and to investigate the clinical relevance of 3D CT. We hypothesized that the results of operative and non-operative treatments would be different in pediatric medial epicondyle fractures with more than 5 mm, as accurately measured by 3D CT.

## 2. Materials and Methods 

This study was approved by the institutional review board of the Gangnam Severance Hospital, Seoul, Korea (25 September 2019; 3-2019-0242). We performed a retrospective chart review of pediatric patients treated for medial epicondyle fracture between January 2007 and December 2018 at our hospital. The inclusion criteria were as follows: (1) isolated medial epicondylar fracture without concomitant elbow dislocation, (2) presence of ossification on the medial epicondyle, (3) fracture displacement >5 mm on 3D CT, and (4) minimum follow-up time of 3 months. Patients were excluded for the following reasons: another upper extremity fracture, a history of elbow surgery or deformity, insufficient medical records, and inadequate preoperative 3D CT or follow-up radiographs available for review. We excluded patients with absolute surgical indications such as incarceration of fracture fragment, open fracture, or ulnar nerve entrapment. Subjects who did not complete the telephone survey were also excluded.

We initially identified 168 patients who were treated for medial epicondyle fractures with displacement of >5 mm. Forty patients were excluded for being associated with elbow dislocation. Among them, three had incarceration of fracture fragment and two had evidence of ulnar nerve entrapment. In addition, 21 patients were further excluded as they did not have preoperative 3D CT scans or had incomplete radiographs. Among the remaining 107 patients, 30 patients were excluded for not having reliable contact information and not being available for the telephone survey. 

A total of 77 patients (50 boys and 27 girls) were included in this study. Forty-five patients were involved in the dominant arm. The mean age at the time of injury was 11.4 years. (range 7–15 years). The mean radiological follow-up duration was 7.5 months (range 3–23 months). The mean functional follow-up period for the telephone survey was 40 months (range 6–70 months). 

**Figure 1:**
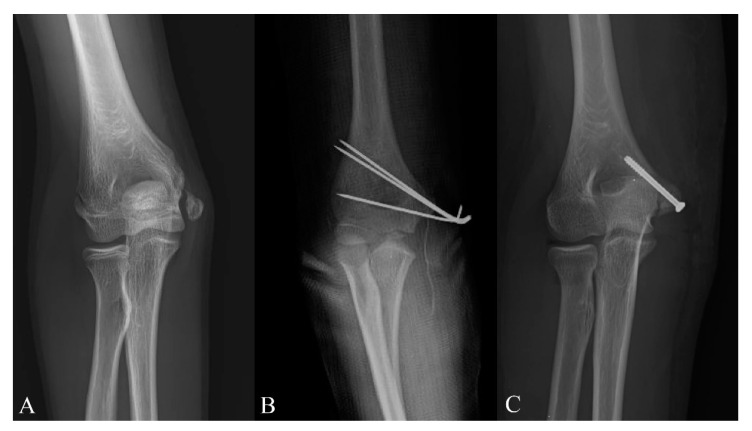
(**A**) Fracture of the right medial epicondyle with marked displacement in an 11-year-old boy, treated with cast immobilization only. (**B**) Displaced fracture of the right medial humeral epicondyle in an 11-year-old boy, treated with open reduction and internal fixation with Kirschner’s wire. (**C**) Displaced fracture of the right medial humeral epicondyle fixed with one cannulated screw in a 11-year-old girl.

Five surgeons were involved in this study, and the choice of treatment modality was decided based on the surgeon’s preference. Patients were divided into the two groups according to the treatment method. Twenty-one patients (Figure 1A) underwent nonoperative treatment; nonoperative management consisted of immobilization of the elbow in a long arm cast at 80° to 90° of elbow flexion and neutral forearm rotation for 3 to 5 weeks. Fifty-six patients underwent surgical treatment, and they were divided into the following two subgroups according to the fixation method: 31 patients (Figure 1B) were treated with open reduction and internal fixation with K-wires followed by immobilization for 4 to 6 weeks. K-wires were removed at 4 weeks after surgery. Twenty-five patients (Figure 1C) had undergone open reduction and internal fixation with screws, followed by immobilization for 2 to 4 weeks. One cannulated screw without washer was used in all patients except for one patient with fragment breakage. Screws were removed if there were irritation symptoms from screw prominence or because of the patients’ demands at least 6 months after surgery.

The following data were obtained: age at injury, sex, amount of displacement, treatment method, type of implant used, duration of immobilization in a cast, the time to restore full range of motion, postoperative complications, and subsequent surgery. 

**Figure 2:**
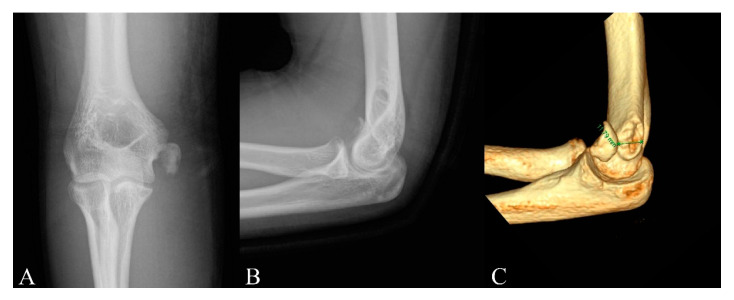
(**A**) Anteroposterior radiograph of a 15-year-old boy with a displaced medial epicondyle fracture. (**B**) Lateral radiograph demonstrates no substantial displacement of the medial epicondyle of the humerus in the same patient. (**C**) Three-dimensional computed tomography scans demonstrate the anterior displacement of the medial epicondyle.

Three-dimensional CT scans were used to measure the amount of fracture displacement. Three-dimensional reconstructions could provide image detailing of the location of the fragment in relation to its origin. The direction of displacement was assessed by using 3D imaging. Then, direct measurement of displacement in millimeters was obtained on this view (Figure 2). Observers were blinded to all study-related data including patient-identifying data, previous measurements, treatment received, and clinical outcomes. Two blinded observers each made two separate measurements at 2 weeks apart. The maximum amount of displacement was recorded and averaged to assess the amount of fracture displacement.

All patients were routinely observed at 1, 2, and 3 months after surgery. Active range of motion was initiated after cast removal. If the patient had any functional deficit or limitation of movement at 3 months after surgery, we continued to examine the patient at intervals of 1 to 2 months. Physical therapy was initiated at 3 months postoperatively if full range of motion had not yet been achieved. We discharged the patients when full movement was achieved. 

If the fracture had not united after >3 months following conservative or surgical treatment, it was defined as a nonunion. Patients were evaluated for the range of motion (ROM) of the involved elbow, pain over the medial epicondyle, valgus instability, and the presence of cubitus valgus at the latest follow-up examination. The limitation of elbow ROM was defined as the loss of ROM of >5° even at 6 months after surgery. The presence of cubitus valgus was assessed by the clinical carrying angle using a goniometer and the radiographic carrying angle on the latest anteroposterior radiograph [15]. Radiographic carrying angles of the contralateral normal elbow radiographs, which were taken at the patient’s first visit to our institution, were measured for comparison. 

Patients were asked to complete the short version of the Quick DASH outcome questionnaire by telephone survey. This score ranged from 0 to 100, with higher scores representing greater disability [16].

To compare the clinical characteristics and radiologic measurements between the two groups, the two-sample t test was used for continuous variables, and χ^2^ test or Fisher’s exact test was used to compare categorical variables. Inter- and intraobserver reliabilities were also gauged via intraclass correlation coefficients, which are interpreted as follows: poor, <0.4; marginal, >0.4 but <0.75; and good, >0.75. In order to avoid complications, the number-needed-to-treat (NNT) analysis was performed to evaluate the effectiveness of surgery. The level of significance was set at *p* < 0.05. Statistical analyses were performed using SPSS software (version 25.0, SPSS; Chicago, IL, USA). Because of the small number of subjects, a post hoc power calculation was carried out using a G Power test (version 3.1.9.4).

## 3. Results

The characteristics and overall outcomes of the two groups divided by treatment method are summarized in Table 1. The two groups were comparable in terms of the mean age at injury, sex, dominance, the mean amount of fracture displacement, the mean immobilization period, and the mean radiologic and functional follow-up period. Bony union rate was significantly higher in patients who were treated operatively than in those treated non-operatively (96.4% vs. 23.8%, *p* < 0.001). Although the mean time to restore full ROM in the operative group was significantly shorter than in the nonoperative group (9.6 vs. 12.6 weeks, *p* < 0.001), there was no significant difference between the two groups with respect to the proportion of patients with restoration of full ROM, the mean Quick DASH score, and the proportion of patients with cubitus valgus. The power was 1.00 for union rate and 0.99 for time to restore full ROM.

The characteristics and overall outcomes of the two subgroups divided by the fixation method are summarized in Table 2. The two groups were comparable in terms of the mean age at injury, sex, dominance, the mean amount of fracture displacement, and the mean radiologic and functional follow-up period. Although the mean immobilization period and the mean time to restore full ROM in the screw group was significantly shorter compared to the K-wire group (*p* < 0.001 and *p* = 0.008, respectively), there was no significant difference between groups in terms of the union rate, the mean Quick DASH score, the proportion of patients with restoration of full ROM, and the proportion of patients with cubitus valgus. The power was 1.00 for the immobilization period and 0.86 for the time to restore full ROM.

The measurements of displacement on CT showed good intra- and interobserver reliability, with intraclass correlation coefficients ranging from 0.78–0.92. The NNT operatively to prevent one symptomatic nonunion was seven.

**Figure 3:**
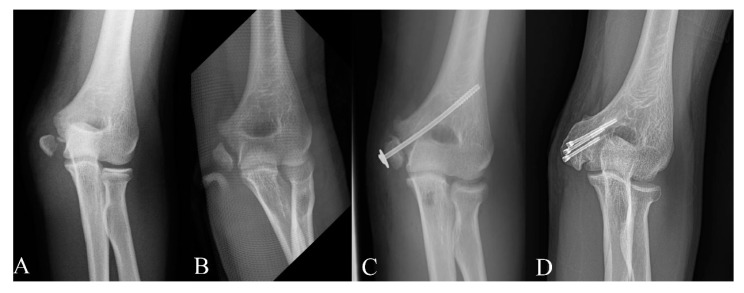
(**A**) Radiograph demonstrating a medial epicondyle fracture with marked displacement in a 13-year-old girl at the initial time of injury. She was treated with cast immobilization only. (**B**) Established medial epicondyle nonunion was seen at 6 months after injury. She had tenderness upon palpation over the medial epicondyle and pain with valgus stress. (**C**) Nonunion was still seen at 1 year after open reduction and internal fixation with a screw and washer. (**D**) Bony union was achieved at 6 months after revisional osteosynthesis with an autologous bone graft.

Complications in each group are summarized in Table 3. In the nonoperative group, the rate of nonunion was 76.2%, and associated complications developed in three patients. Although we found a similar proportion of patients who had limitation of ROM in each group, there were three patients with loss of ROM of >15° in patients who were treated conservatively due to symptomatic nonunion. An 11-year-old female patient underwent osteosynthesis and ulnar nerve anterior transposition. At 2-year follow up, osseous union and stability were obtained with a mild flexion contracture of 10°. Another 13-year-old female patient underwent open reduction and internal fixation with a screw and washer. Bony union was not achieved after 1 year, and the patient still complained of pain and weakness. Revisional osteosynthesis with three cannulated compression screws and an autologous olecranon bone graft were performed, and radiographic union was achieved at 6 months after surgery (Figure 3). She exhibited a flexion contracture of 20° with no valgus instability at the final follow-up. The remaining 15-year-old male patient underwent excision of the osteocartilaginous fragment, with suture of the tendons and medial collateral ligament to the adjacent periosteum. He felt that elbow was unstable and had a 20° limitation of elbow extension. In the K-wire fixation group, superficial wound infection developed in two patients who were treated with intravenous antibiotics. In the screw group, two patients developed nonunion, but they had no symptoms and a limited range of motion of <15°. Fragmentation of the medial epicondyle occurred in 1 patient, but union was achieved with additional fixation using a small headless screw. Screws were removed in 18 patients at a mean time of 7.3 months postoperatively. 

## 4. Discussion

CT provides a better understanding of the fracture pattern and displacement in pediatric medial epicondylar fracture [12]. It was known that the displacement of fracture may be underestimated by standard AP and lateral views of elbow [13,14]. CT scan may change the treatment approach after precise measurement for the displacement in the fracture that is borderline on plain radiographs or even the one that appears to be less than 5 mm. However, our results showed that there was no difference in functional outcomes between operatively and nonoperatively treated displaced medial epicondyle fractures with displacement of >5 mm, as accurately measured on 3D CT, aligning with results of prior studies using traditional methods of displacement measurement [5,6,7,8,17,18,19]. Although CT scan can be diagnostic in minimal displaced medial epicondylar fracture, performing CT only to measure the fracture displacement may be reconsidered in large displaced fractures, which can be clearly diagnosed with simple radiograph. 

The rate of nonunion was very high in the nonoperatively treated group. The reported rate of nonunion in the nonoperative treatment ranges from 0% to 90% [2,5,19,20,21]. This wide range resulted from the fact that several studies included patients with mild fracture displacement of <5 mm [2,21]. The nonunion rate was reported to be up to 90% to 100% in previous studies conducted in only patients with >5 mm of large displacement [5,22]. This high nonunion rate should be recognized as a result of conservative treatment for large displaced medial epicondyle fractures.

Although symptomatic nonunion of the medial humeral epicondyle is a relatively rare entity, it is known as a challenging complication [23,24]. Few studies have shown that even medial epicondyle fractures with >5 mm of displacement can be managed nonoperatively [5,22]. However, in these old studies, the assessment of treatment outcome was somewhat arbitrary. Most importantly, it was doubtful that there was no symptomatic nonunion in patients who were treated conservatively. In the present study, three patients were symptomatic with loss of elbow extension of more than 15° and joint instability. Those patients should have undergone osteosynthesis and had some degrees of fixed flexion contracture even after surgery. Moreover, some of the other patients showing nonunion may develop symptoms later in life. A case study reported the long-standing symptomatic nonunion of the medial epicondyle in the adult population [23] for those whose injuries occurred in childhood. It should be noted that symptomatic nonunion could be a potential problem.

Surgical treatment was optimal to achieve the bone union in all patients. Unlike osteosynthesis for symptomatic nonunion [23], the surgery for medial epicondyle fracture is simple. However, surgical treatment may not be cost-effective, and the NNT operatively to prevent one symptomatic nonunion was seven. Although implant removal is not mandatory, the overall cost of surgical treatment, including implant removal, is usually high [25].

There was no difference in the proportion of patients with restoration of full ROM between the two groups divided by treatment method. Other comparative studies have also shown no difference in the recovery rate of ROM [2,5,9]. However, in the present study, nonoperative patients tended to achieve full ROM faster than operative patients did. This result seems to support the postoperative elbow stiffness due to the combination of surgical scarring of capsule and periarticular ligament structures along with postoperative immobilization [6,11]. 

Both K-wire and screw yielded similar radiologic and functional outcomes. Although fixation with screw allows for rigid fixation and early mobilization [1,25], it can cause some complications such as penetration and fragmentation of the epicondyle [3]. There was one case of fragmentation in patients treated with screw fixation. However, K-wire fixation is less stable, and supplementary cast immobilization is also required [3,25]. We also found that the average immobilization period and the mean time to restore full ROM was longer in patients treated with K-wire. One study suggested that immobilization for over 2 weeks was associated with a statistically significant loss of range of motion [26], but, in our study, there was no difference in the proportion of patients with restoration of full ROM between the K-wire group and the screw group. This discrepancy may have resulted from the fact that all subjects in the present study had isolated medial epicondylar fractures without elbow dislocation. Although the best surgical treatment option for this injury seems to achieve stability and allow early mobilization, surgeons should consider the pros and cons of each fixation device when selecting the fixation method.

Cubitus valgus is one of the complications in medial epicondyle fractures. This complication has been described in only a few previous reports [5]. We postulated that cubitus valgus resulted from relatively elongated medial collateral ligament due to displaced fragment with fibrous union or malunion. Nevertheless, none of the patients with cubitus valgus had any functional impairment since the degree of cubitus valgus was <10° compared to the contralateral side. 

Our study had several limitations. First, this was a retrospective study without randomization and it had several flaws, including incomplete data, the retrospective application of functional outcome scores, and the fact that the decision for operative versus nonoperative management was made based on the preference of the treating physician. Although five surgeons were involved in this study, the operative technique and post-operative protocols were similar among surgeons. Second, telephone surveys were used to evaluate patients functionally. Therefore, clinical data such as objective range of motion measurement, physician assessment of deformity, and up-to-date radiographs were not available, prohibiting us from commenting in detail on these outcomes. Lastly, the long-term follow-up for all patients has not been investigated in the present study. The occurrence of symptomatic nonunion or cubitus valgus may not be properly investigated due to short term follow-up periods in some patients. Larger prospective studies are needed to draw a more definitive conclusion.

## 5. Conclusions

In a pediatric medial epicondylar fracture with displacement of >5 mm as accurately measured on 3D CT, there was no difference in functional outcomes between operative and nonoperative treatment. The bony union rate was much higher in patients treated surgically, which was not correlated with functional outcomes. The fixation method does not seem to affect surgical treatment outcomes. Treatment should be individualized, with consideration of the risk of symptomatic nonunion and medical cost for surgery. Performing CT only to measure the fracture displacement in obviously displaced medial epicondylar fracture is not considered as a part of the “necessary” diagnostic workups.

## Figures and Tables

**Table 1:** Comparison between the groups by treatment method.

Variables	Nonoperative (*n* = 21)	Operative (*n* = 56)	*p*-Value
Age at injury, year	10.8 (7 to 15)	11.6 (7 to 15)	0.218
Sex, *n* (%)			0.380
Male	12 (57.1)	38 (67.9)	
Female	9 (42.9)	18 (32.1)	
Dominant arm, *n* (%)	13 (61.9)	32 (57.1)	0.706
Fracture displacement, mm (range)	8.7 (6 to 15)	9.6 (5 to 20)	0.262
Immobilization period, week (range)	4.1 (3 to 5)	4.5 (2 to 6)	0.118
Radiological follow-up period, month	7.6 (3 to 23)	7.4 (3 to 20)	0.884
Union, *n* (%)	5 (23.8)	54 (96.4)	<0.001
Quick DASH score	1.3 (0 to 9.08)	0.6 (0 to 9.08)	0.169
Score = 0, *n* (%)	17 (81)	47 (84)	
Score > 0, *n* (%)	4 (19)	9 (16)	
Functional follow-up period for phone survey, month	36.6 (6 to 70)	40.1 (6 to 70)	0.456
Patients with restoration of full ROM, *n* (%)	16 (76.2)	46 (82.1)	0.557
Time to restore full ROM, week	9.6 (6 to 12)	12.6 (7 to 20)	<0.001
Within 3 months, *n* (%)	16 (100)	30 (65.2)	
Cubitus valgus, *n* (%)	4 (19)	3 (5.4)	0.063

Values are given as mean (range) unless otherwise indicated.

**Table 2:** Comparison between the groups by fixation method.

Variables	K-Wire (*n* = 31)	Screw (*n* = 25)	*p*-Value
Age at injury, year	11.1 (7 to 14)	12.3 (8 to 15)	0.083
Sex, *n* (%)			0.081
Male	18 (57.1)	20 (80)	
Female	13 (42.9)	5 (20)	
Dominant arm, *n* (%)	17 (54.8)	15 (60)	0.698
Fracture displacement, mm (range)	9.4 (5 to 14)	10.1 (5 to 20)	0.496
Immobilization period, week (range)	5.6 (4 to 6)	3.3 (2 to 4)	<0.001
Radiological follow-up period, month	6.8 (3 to 20)	8.1 (3 to 18)	0.232
Quick DASH score	0.4 (0 to 4.50)	0.7 (0 to 9.08)	0.502
Score = 0, *n* (%)	27 (87)	20 (80)	
Score > 0, *n* (%)	4 (13)	5 (20)	
Functional follow-up period for phone survey, month	45.2 (6 to 70)	36.9 (6 to 70)	0.196
Patients with restoration of full ROM, *n* (%)	26 (83.9)	20 (80)	0.707
Time to restore full ROM, week	13.5 (8 to 20)	11.3 (7 to 16)	0.008
Within 3 months, *n* (%)	14 (53.8)	16 (80)	
Cubitus valgus, *n* (%)	1 (3.2)	2 (8)	0.430

Values are given as mean ± standard deviation unless otherwise indicated.

**Table 3:** Summary of complications among the groups.

Variables	Nonoperative (*n* = 21)	K-Wire (*n* = 31)	Screw (*n* = 25)
Nonunion, *n* (%)	16 (76.2)	0 (0)	2 (8)
Limitation of full ROM, *n*	5	5	5
<15 degrees	2	5	5
>15 degrees	3	0	0
Symptomatic nonunion with valgus instability, *n*	3	0	0
Cubitus valgus, *n*	4	1	2
Superficial wound infection, *n*	N/A	2	0
Fragment breakage, *n*	N/A	0	1
Subsequent surgery, *n*			
Osteosynthesis	2	0	0
Excision of bony fragment	1	0	0
Removal of hardware	0	0	18
